# Development and validation of a quantitative real-time polymerase chain assay for universal detection of the White Spot Syndrome Virus in marine crustaceans

**DOI:** 10.1186/1743-422X-10-186

**Published:** 2013-06-07

**Authors:** Fernando Mendoza-Cano, Arturo Sánchez-Paz

**Affiliations:** 1 Laboratorio de Referencia, Análisis y Diagnóstico en Sanidad Acuícola. Centro de Investigaciones Biológicas del Noroeste S. C. (CIBNOR), Calle Hermosa 101. Col. Los Ángeles., Hermosillo. Son C.P. 83106, México

**Keywords:** WSSV, Quantitative PCR, VP28, Specificity, Diagnosis, Universal primers

## Abstract

**Background:**

The White Spot Syndrome Virus (WSSV), the sole member of the family Whispoviridae, is the etiological agent that causes severe mortality events in wild and farmed shrimp globally. Given its adverse effects, the WSSV has been included in the list of notifiable diseases of the Office of International Epizootic (OIE) since 1997. To date there are no known therapeutic treatments available against this lethal virus, and a surveillance program in brood-stock and larvae, based on appropriate diagnostic tests, has been strongly recommended. However, some currently used procedures intended for diagnosis of WSSV may be particularly susceptible to generate spurious results harmfully impacting the shrimp farming industry.

**Methods:**

In this study, a sensitive one-step SYBR green-based real-time PCR (qPCR) for the detection and quantitation of WSSV was developed. The method was tested against several WSSV infected crustacean species and on samples that were previously diagnosed as being positive for WSSV from different geographical locations.

**Results:**

A universal primer set for targeting the WSSV VP28 gene was designed. This method demonstrated its specificity and sensitivity for detection of WSSV, with detection limits of 12 copies per sample, comparable with the results obtained by other protocols. Furthermore, the primers designed in the present study were shown to exclusively amplify the targeted WSSV VP28 fragment, and successfully detected the virus in different samples regardless of their geographical origin. In addition, the presence of WSSV in several species of crustaceans, including both naturally and experimentally infected, were successfully detected by this method.

**Conclusion:**

The designed qPCR assay here is highly specific and displayed high sensitivity. Furthermore, this assay is universal as it allows the detection of WSSV from different geographic locations and in several crustacean species that may serve as potential vectors. Clearly, in many low-income import-dependent nations, where the growth of shrimp farming industries has been impressive, there is a demand for cost-effective diagnostic tools. This study may become an alternative molecular tool for a less expensive, rapid and efficient detection of WSSV.

## Background

The White Spot Syndrome Virus (WSSV) is an extremely lethal and contagious shrimp pathogen. It has emerged globally as the major threat for shrimp farming during the last decades as outbreaks of WSSV lead to cumulative mortalities of 100% within 3–10 days after the onset of clinical signs [1,2]. WSSV was first detected in Taiwan in 1992 [2], and since then it has spread globally to all major shrimp farming areas with considerable socio-economic consequences. To date, several studies have shown that there are both multiple routes of transmission and multiple potential host reservoirs (more than 90 species of arthropods and some carriers belonging to other phyla that may constitute a potential source of infection) for WSSV [3]. Thus, the disease caused by WSSV has been listed as notifiable by the Office of International Epizootic (OIE) Aquatic Animal Health Code since 1997 [4].

Nowadays, there is no known therapeutic treatment available to prevent or reduce the adverse effects of WSSV, and conducting a routine and intensive surveillance program in both brood-stock and larvae, based on appropriate diagnostic tests, as an effective strategy to prevent the occurrence of outbreaks in shrimp farming facilities, has been recommended [5,6].

Currently, several methods have been established to detect WSSV, each having individual benefits and disadvantages which should be considered in their application. The main methods reported for WSSV detection include: 1) the use of monoclonal and polyclonal antibodies [7-13], which has been described as simple, inexpensive and provides results considerably fast [14], but this method may be hampered by its low sensitivity [14,15]; 2) the use of biosensors [16-18], which have demonstrated a remarkably high sensitivity and a less elaborated sample preparation procedure. However, commercially available biosensors are often very expensive and require instrument-specific consumables [19]; 3) non-PCR molecular methods for WSSV DNA amplification [20-23], which, due to its basic principles of using four to six primers that recognize six to eight areas of the target sequence and the usage of the *Bst* DNA polymerase, offers the advantage of a high sensitivity and the synthesis of large amounts of amplicons under isothermal conditions. Furthermore, as less manipulation of samples is required, it has been suggested that the risks of accidental contamination can be significantly reduced [23]. However, one of the most important factors in optimizing these methods relies on the design of appropriate primers, a more complex procedure than that for conventional PCR [24,25], and 4) and conventional PCR, which is generally considered as the method of choice for the detection of viral DNA present at very low amounts in biological samples [4,26-31], but, just as the above mentioned methods, a number of important disadvantages have been reported by the use PCR for WSSV detection. Thus, the Office of International Epizootic (OIE) recommends, among few other molecular techniques, the use of a two-step nested PCR protocol described by [26,32] for all situations where WSSV diagnosis is required [33]. However, [34] suggested for the first time the possibility that this protocol may lead to some false-negative results, and subsequently [29] reported that due to its low annealing temperature (55°C) this method lacks specificity as it generates false-positive results. Furthermore, sequence analysis of the amplicon obtained showed no significant phylogenetic relationship to WSSV. To overcome these limitations, extensive modifications have been developed to give more accurate results. Thus, [4] have recently reported a modified protocol, substantially faster and as sensitive of the two-step nested PCR reported by [26]. Finally, a number of variations on the basic theme of PCR have been developed. Several studies have shown the successful use of real-time PCR (qPCR) assays to detect, and quantify, the viral load of WSSV infected organisms [35-40]; however, all of these methods depend on the use of sequence-specific oligonucleotide hydrolysis probes, which are a more expensive option than using SYBR green chemistry [41]. Thus, a sensitive SYBR Green-based qPCR assay may be particularly advantageous in developing nations where shrimp farming, an activity of great economic and social importance, has seen an impressive growth in recent years [42].

Furthermore, some studies have previously demonstrated that considerable genetic variability exists among WSSV natural isolates. Accordingly, the possible occurrence of WSSV variants infecting exclusively non-shrimp hosts has been reported [34-43]. In addition, [44] and [45] found striking differences on the genome size of WSSV isolates from different shrimp species, which are implied on the differential virulence of WSSV isolates [44]. Finally, a recent study showed the presence of two WSSV genetic variants in Vietnam, which may have arisen on different occasions by recombination of isolates introduced by human activities. On the other hand, the possible existence of certain degree of genetic diversity within WSSV populations in this geographic area cannot be excluded to explain this result [46]. Thus, the diversity and geographic distribution of WSSV genetic variants increases the possibility of diagnostic failures, which may lead to dispersal of the virus.

Considering that no consensus has been reached so far over the optimal method to detect WSSV, and as there are no reports of a molecular assay capable to universally detect this virus in crustaceans, there is need to develop an efficient, single step and sensitive PCR-based method, designed from appropriate DNA target sequences. The data obtained in the present study shows the successful development of a rapid, sensitive and accurate PCR-based procedure for the universal detection of WSSV in different crustaceans.

## Results and discussion

### Primer design and PCR amplification

Based on a multiple sequence alignment the PCR primers evaluated in this study were designed based on a highly conserved region of the WSSV VP28 gene. VP28, the most abundant exposed protein in the WSSV envelope, is encoded by the open reading frame (ORF) 421 (*wsv421*) [47], and the resulting protein contains 204 amino acid residues with a theoretical molecular mass of 22 kDa [48]. It is thought that WSSV VP28 plays a critical role during early events of virus infection, particularly as a viral attachment protein [49], and that it may contribute importantly to the recognition of receptors at the shrimp cell surface [48]. The crystal structure of VP28 consists of 12 copies of the protein assembled into four trimers, each monomer exhibiting a single β-barrel and a α-helix protruding from the β-barrel architecture, and the predicted N-terminal transmembrane region of VP28 may anchor on the viral envelope membrane [50]. Thus, due its essential role and the conserved nature of the gene encoding VP28 among several WSSV geographical isolates it seems as an appropriately sensitive target for PCR diagnosis in crustaceans.

**Figure 1:**
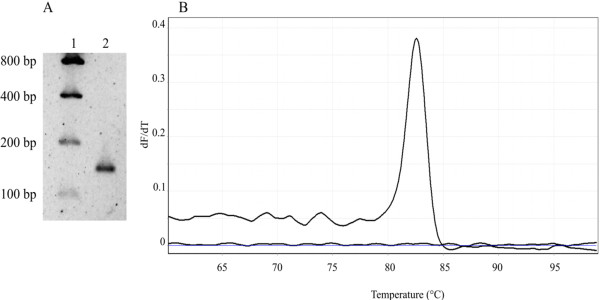
**One-step PCR amplification of a 141-bp fragment of the WSSV VP28 encoding gene.** (**A**) The ethidium bromide-stained amplification product of WSSV VP28 generated by using the VP28-140Fw and VP28-140Rv primers was electrophoresed on a 1.8% agarose gel. Lane 1: low DNA mass ladder; lane 2: 141-bp PCR amplification product of the WSSV VP28 gene. (**B**) Melting curve of real-time PCR for a single WSSV VP28 fragment product with a melting temperature of 82.63°C.

Standard PCR using the VP28-140Fw and VP28-140Rv primers revealed a single amplicon of 141 bp in size, after agarose gel electrophoresis (Figure 1A), and subsequent melting analysis by qPCR showed a single 82.63°C melting domain (Figure 1B) indicating a specific amplification product. Sequence analysis of the amplicon using BLAST showed 100% homology with several WSSV VP28 gene sequences deposited in GenBank. This sequence encodes a peptide (VWNNTSRKINITGMQMVPKINPSKAFVGSSNTSSFTPVSIDEDEVG) corresponding to a segment of the protrusion emanating outside the viral envelope, which has been suggested to exert an essential role in the initial interaction with host receptors [50]. Due to its probable essential role in mediating viral entry, amino acid changes on the structure of envelope proteins may alter the binding affinity for host receptors compromising viral fitness. Thus, few changes may be expected to occur naturally in this protein, providing unique regions that will aid in the universal diagnosis and monitoring of this lethal virus.

### Primer specificity and sensitivity

The *in silico* analysis of the VP28-140Fw and VP28-140Rv primers demonstrated its specificity for detection of WSSV. As expected, best BLAST hits (low expectation values, E-values) were found for nucleotide sequences of the genome of the WSSV virus while high E-values (9×10^-7^-4x10^-6^) were obtained in short nucleotide sequences (5–8 nt) on the genome of the viruses studied. Primer sequences with low E-values are more similar to the WSSV VP28 gene, and virtually identical short alignments of other viruses showed high E-values because its calculation is based on the length of the query sequence, and shorter sequences have higher probabilities of occurring in the database purely by chance. Thus, the high target specificity of the primers designed in this study was assured by avoiding significant cross-homologies among its sequences and those of other shrimp infecting viruses.

No cross-reactivity was observed when a panel of 6 known positive and negative samples of shrimp infecting viruses were included, indicating that the specificity and sensitivity of the assay was 100% (data not shown). Similar results have been obtained by using the nested-PCR approach previously described [32], a subsequent modification of it [4], or by an immunodot assay test [51]. However, it must be underlined that it has been suggested that the two-step PCR procedure for detection of WSSV [32], still considered a useful and reliable PCR test for diagnosis of WSSV by the OIE, lacks specificity [29] as its low annealing temperature (55°C) may generate false positives which, after sequence analysis, showed not to be part of the WSSV genome [4]. In the present study, more stringent PCR conditions were enforced for the detection of WSSV than those applied previously [32], providing thus greater specificity.

### Standard curve for qPCR and limit of detection

**Figure 2:**
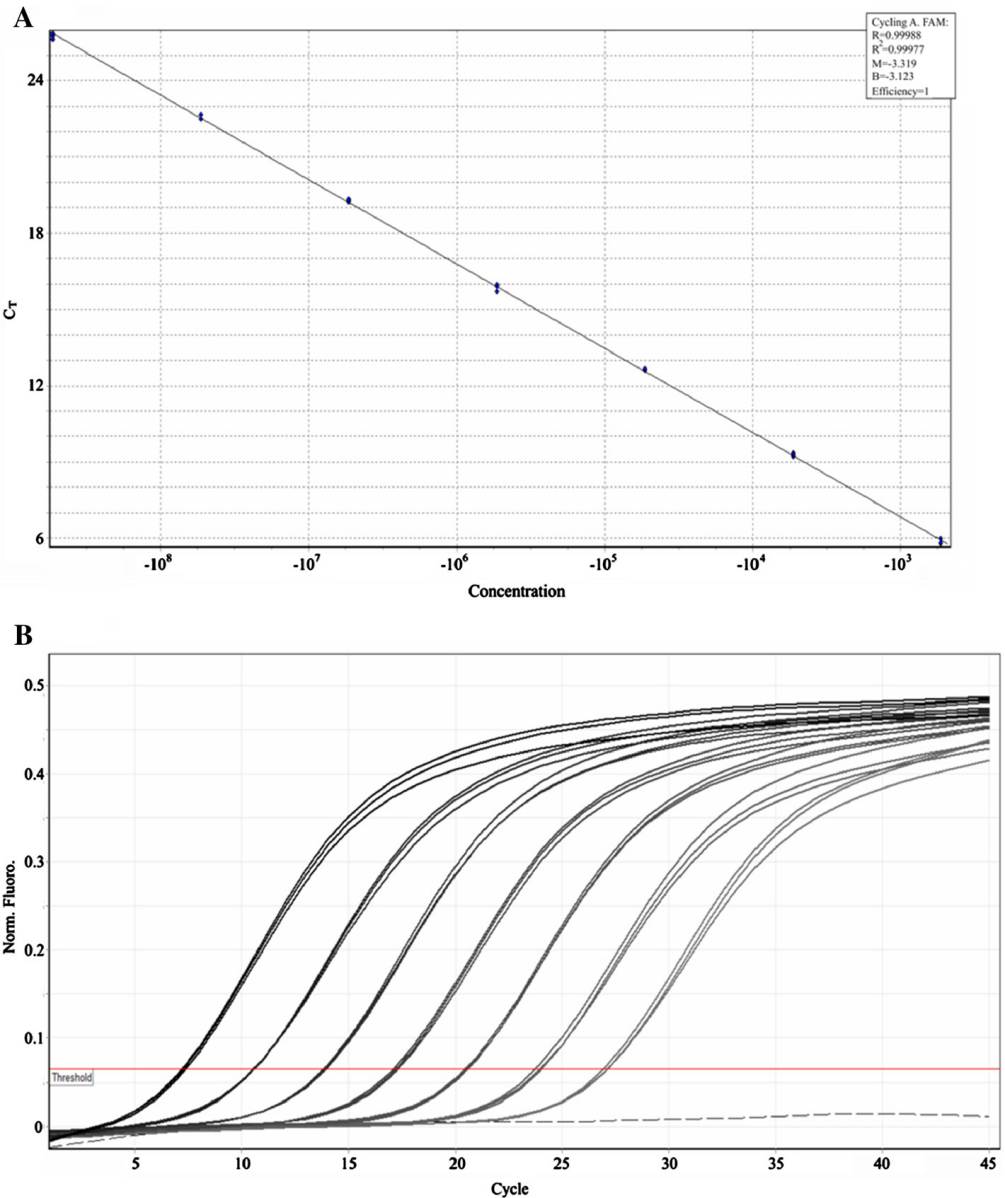
**Quantitative real-time PCR standard curve for a 141 bp WSSV VP28 fragment.** (**A**) Standard curve for real-time PCR. A ten-fold serially diluted 141 bp WSSV VP28 DNA fragment was amplified and analyzed in real-time (1.24 x 10^8^ to 1.24 x 10^2^ copies/μL). Quantification cycle (C_q_) values were plotted against copy number to construct the standard curve. R^2^=0.999, and amplification efficiency (E) =1. (**B**) Representative amplification plot for WSSV VP28 DNA fragment dilutions.

Figure 2A shows the linear range of the standard curve of the WSSV VP28 fragment. The standard curve generated was linear over a range of 7 log units. The upper and lower quantification limits were 1.24 × 10^7^ and 12 copies per PCR reaction, respectively. This detection limit is as low as that reported by using a nested PCR protocol [32], a fluorescent quantitative PCR protocol [52] or a loop-mediated isothermal amplification method [21], and more sensitive than the nested PCR protocol previously reported [53] (which may require a minimum of 1000 copies [54]).

### Efficiency, linearity and precision

The qPCR standard curve showed evenly distributed quantification cycle values (~3.1 cycles), with strong linear correlations between the quantification cycles (C_q_) values and the log of the input template DNA amount (Figure 2B). Thus, considering that the efficiency of a PCR reaction is calculated from the slope of the standard curve (according to the equation: Log E = 10^(−1/slope)-1^), this assay showed a high amplification efficiency (ranging from 98 to 101%).

Furthermore, the correlation coefficient (r or R^2^) value of a standard curve represents how closely the experimental data fit the regression line, or how linear the data are. Linearity, in turn, gives a measure of the variability across assay replicates, and whether the amplification efficiency is the same for different starting template copy numbers. A significant difference in observed C_q_ values between replicates will lower the *r* or *R*^2^ value [55]. In this study, *R*^2^ values ranged from 0.98 to 0.99 (Figure 2A), indicating high linearity and, thus, an adequate quantification capacity of the reactions. Similar results have been reported by the use of other methods as biosensors, which showed linearity ranging from 1 to 10^6^ copies [18,56,57].

**Figure 3:**
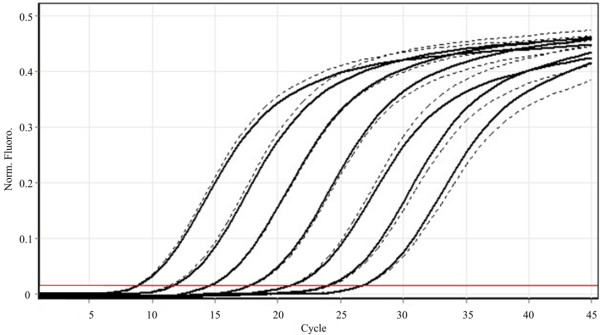
**Performance comparison of two different commercial kits for WSSV detection by qPCR.** The solid line represents iQ SYBR® Green supermix (Biorad) amplification, whereas the dashed line represents amplification using GoTaq qPCR Master mix (Promega). No differences were detected.

To determine the reproducibility (precision, according to [58]) of the qPCR assay, aliquots of 7 serial dilutions of the stock DNA (Sonora sample) were assayed in triplicate two times over a 2 months period, and it was determined by calculating the coefficient of variation (CV) for each sample mean copy number. CV for replicate measurements ranged from 3.44% to 10.4%, with a median value of 6.13%. Furthermore, the GoTaq® qPCR Master mix was used as an alternative to the iQ SYBR® Green supermix, and similar results were obtained with both approaches (Figure 3).

### Amplification of WSSV samples from different geographical regions

**Figure 4:**
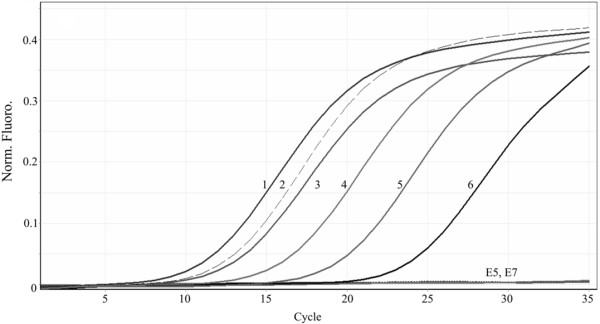
**Detection of WSSV in different geographical isolates and precision of the assay. 1**: Madagascar sample (3.2x10^7^ copies/μL), **2**: Sonora sample (1.38x10^6^ copies/μL), **3**: University of Arizona sample (1.25x10^6^ copies/μL), **4**: Saudi Arabia sample (2.47x10^5^ copies/μL), **5**: China sample (5.6x10^3^ copies/μL), **6**: Brazil sample (4.5x10^3^ copies/μL), E5 and E7, SPF DNA samples from Brazil. WSSV was detected in all infected samples.

To our knowledge, there is only one previous report in which samples from different geographical locations (Indonesia, China, Nicaragua, Honduras, Brazil and US) were successfully detected [4]. The primers designed in the present study were shown to exclusively amplify the targeted WSSV VP28 fragment, and successfully detected the virus in different samples regardless of their geographical origin (Brazil, China, Madagascar, Saudi Arabia and Mexico) (Figure 4).

### Detection of WSSV on a wide range of crustaceans

**Figure 5:**
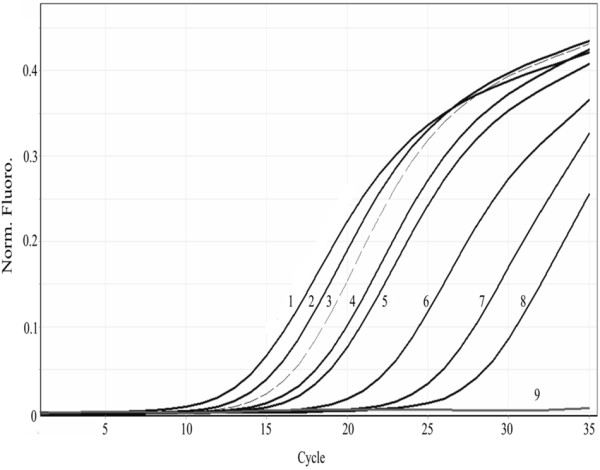
**WSSV detection in different crustacean species. 1**: *Macrobrachium rosenbergii* (3.4x10^7^ copies/μL) , **2**: *Petrolisthes edwardsii* (2.59x10^7^ copies/μL), **3**: *Penaeus vannamei* (Sonora sample) (1.28x10^6^ copies/μL), **4**: *Calanus pacificus californicus* (6.1x10^5^ copies/μL), **5**: *Lysmata californica* (5.4x10^5^ copies/μL), **6**: *Penaeus stylirostris* (2.13x10^4^ copies/μL), **7**: *Callinectes bellicosus* (5.98x10^3^ copies/μL), **8**: *Palaemon ritteri* (3.63x10^3^ copies/μL), and **9**: Negative control (SPF DNA).

A further advantage of this method is its ability to successfully detect the presence of WSSV in several species of crustaceans, including both naturally and experimentally infected (Figure 5).

It has been well documented that viruses depend heavily on their host cellular machinery to support its replication, and consequently some constraints, as genomic nucleotide composition, are important factors influencing variations of synonymous codon usage in the WSSV genome [59]. Codon usage preference of viruses may co-evolve with that of their hosts to increase their fitness [60,61]. Codon usage bias, the non-uniform usage of synonymous codons, varies widely between species, possibly between different tissues [62] and, in some cases, between different regions of a genome in a single species [63]. As several studies have demonstrated that WSSV is capable to infect a wide range of crustaceans, including shrimp, crabs, crayfish, lobsters, and copepods [3], variations in the codon usage bias among WSSV isolates from different hosts may be expected.

To the best of our knowledge, the only previous study of WSSV detection in different crustacean species was limited to a few penaeid shrimp species (*Penaeus monodon*, *P. setiferus*, *P. vannamei*, *P. duorarum* and *P. aztecus*) [4], while by using the PCR method developed in this study the presence of WSSV in a number of penaeid and non-penaeid crustacean species (*P. stylirostris*, *P. vannamei*, *Farfantepenaeus paulensis*, *F. brasiliensis*, *P. monodon*, *P. indicus*, *Lysmata californica*, *Palaemon ritteri*, *Calanus pacificus*, and *Macrobrachium rosenbergii,* and *Petrolisthes edwardsii*) was detected*.* Thus, the development of a universal approach for the detection of low WSSV loads in several crustacean species based on the amplification of a highly conserved region of VP28, which carries out an essential role in the systemic infection of crustaceans by WSSV, may prove to be particularly useful.

## Conclusions

WSSV is considered the most devastating viral disease of penaeid shrimp populations, and because of its wide geographic distribution and broad host range, routine surveillance programs, based on appropriate diagnostic tests designed to detect WSSV even in low numbers, are highly important. Because of its negative impact in populations of wild and farmed shrimp, and in other crustaceans, OIE has recommended a number of diagnostic methods for its detection, including both non-commercial PCR protocols [32] and a couple of OIE-registered commercial kits. However, some of these approaches have been subject to criticism because of its lack of specificity [4,29], and modifications have been proposed to improve its performance [4].

The primer set proposed in this study facilitates a reliable protocol for the detection of WSSV, which is critical for the management of the disease. The sensitivity and specificity of this qPCR assay compares favorably with those obtained by using other approaches. Furthermore, the current protocol was used successfully to detect representative WSSV isolates from different geographic regions of the world in a one-step qPCR assay. This is, to our knowledge, the first universal qPCR validated protocol able to detect WSSV isolates despite genomic variations. In addition, the qPCR procedure described here proved to be particularly useful to quantify the WSSV load in samples of a diverse range of decapod crustacean hosts. Finally, an important benefit of the method described in this study is that it is an interesting and a reliable, cost-effective option for the diagnosis of WSSV to implement in nations with limited availability of resources.

## Methods

### Primer design

**Figure 6:**
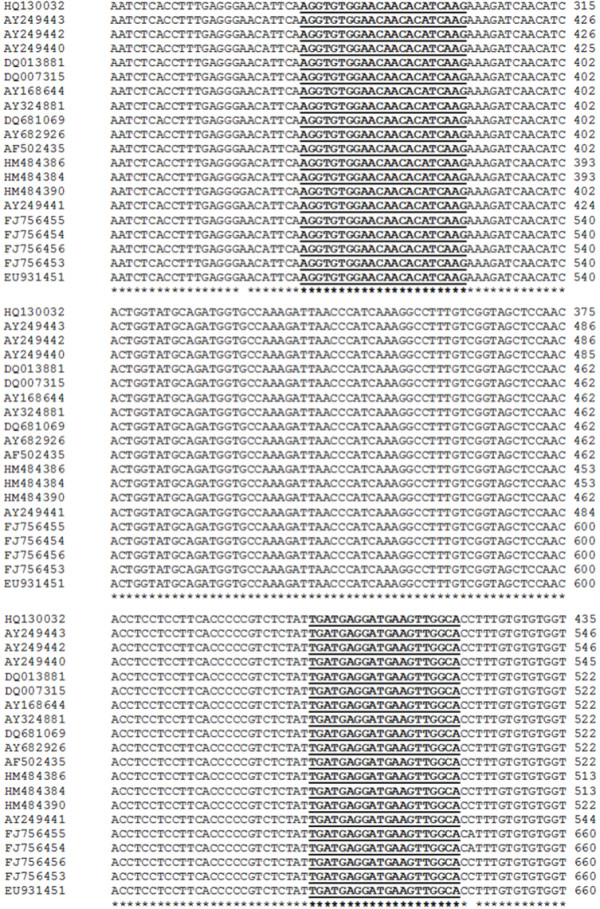
**Alignment of the nucleotide sequences of a WSSV VP28 fragment encoding gene from representative geographic isolates.** Nucleotides were aligned using ClustalW. Primers are shown in bold and underlined. Identical nucleotides are indicated by asterisks. GenBank accession numbers are defined in Table 1.

Primers were designed upon alignment of 20 nucleotide sequences (Table 1, Figure 6) of the WSSV VP28 encoding gene available in the GenBank database of the WSSV. A sequence alignment was generated by using the EMBL-EBI ClustalW (1.82) Multiple Sequence Alignment Tool [64] (at: http://www.ebi.ac.uk/Tools/msa/clustalw2/). PCR primers were strategically designed by the Primer3 software [65] (at: http://frodo.wi.mit.edu/primer3/) to amplify a 140-bp fragment within a highly conserved region of the WSSV VP28 gene. The sequences of the primers were as follows: VP28-140Fw (5′-AGG-TGT-GGA-ACA-ACA-CAT-CAA-G-3′) 1 μL of primer VP28-140Rv (5′-TGC-CAA-CTT-CAT-CCT-CAT-CA-3′) and were synthesized at the oligonucleotide synthesis facility of the Instituto de Biotecnología, UNAM.

**Table 1:** List of the WSSV VP28 DNA sequences used for primer design

**Isolate name**	**Country**	**GenBank Acc. No.**
Unknown	Brazil	HQ130032
Unknown	China/GuangXi	AY682926
China 99/Qindao	China	AY249440
Xiang Shan	China	DQ007315
Chidambaram M8	India/Chidambaram	HM484386
Chidambaram M6	India/Chidambaram	HM484384
Kadalur NM4	India/Kadular reef	HM484390
Unknown	India	DQ681069
Indian	India	DQ013881
Indonesia 97	Indonesia	AY249441
Japan 98	Japan	AY249443
Korea 01	Korea	AY324881
WSSV-Mx-H-2004	Mexico/Sinaloa	FJ756455
Unknown	Mexico/Sinaloa	FJ756456
WSSV-Mx-G-2004	Mexico/Sinaloa	FJ756454
WSSV-Mx-C-2005	Mexico/Sonora	FJ756453
WSSV-Mx-F-2001	Mexico/Sinaloa	EU931451
Unknown	Unknown	AF502435
US 98/South Carolina	USA	AY249442
Unknown	Vietnam	AY168644

### Viral standards

Shrimp tissue samples from Madagascar (MDG), Saudi Arabia (SA) and China (CHN) previously diagnosed as WSSV-positive by the methodology reported by [4] were kindly provided by Dr. Donald Lightner (Department of Veterinary Science and Microbiology, University of Arizona). Three shrimp DNA samples (SJN7B, G7 and B22) diagnosed as WSSV-positive by the nested PCR protocol described by [32] and two SPF DNA samples (E5 and E7) from Brazil (Rio Grande do Sul and Lagoa dos Patos), were gently provided by Dr. Lissandra Souto Cavalli (Laboratório de Virologia Animal, Instituto de Pesquisas Veterinárias Desidério Finamor). DNA samples, diagnosed as WSSV-positive by using the IQ Real WSSV Quantitative System (Farming IntelliGene Tech), of the following WSSV-experimentally infected organisms were provided by Dr. Jorge Hernández (Laboratorio de Referencia, Análisis y Diagnóstico en Sanidad Acuícola, Centro de Investigaciones Biológicas del Noroeste S. C.): blue shrimp (*Penaeus stylirostris*), marine shrimp (*Lysmata californica*), tidepool shrimp (*Palaemon ritteri*), calanoid copepod (*Calanus pacificus*), and freshwater prawn (*Macrobrachium rosenbergii*). In addition, DNA was isolated from WSSV-infected shrimp (*Penaeus vannamei*) collected in a shrimp farming facility located in Sonora, Mexico (named Sonora sample).

DNA from WSSV-infected tissues was isolated using the GeneClean® spin glassmilk solution (MP Biomedicals) following the manufacturer’s instructions. Briefly, up to 100 mg of WSSV-infected tissue were homogenized in 300 μL of lysis buffer (100 mM NaCl, 50 mM Tris pH 8.0, 100 mM EDTA pH 8.0, 1% SDS) by using a Teflon pestle (one stroke, 15 seconds), and immediately centrifuged at 6,000 × g during 10 min. The upper phase (50 μL) was recovered and added to 150 μL of the GeneClean silica matrix, rested for 5 min at room temperature, vortexed and centrifuged at 6, 000 × g for 5 min. Samples were washed by adding 500 μL of washing buffer (a solution of 50% ethanol using 1× TBE) and centrifuged at 6,000 × g during 5 min. This washing step was repeated once more to ensure complete removal of residual contaminants. Finally, samples were air dried for 10 min, and nucleic acids were recovered in 50 μL of nucleases free water.

The concentration of the DNA samples was measured via UV absorption (260/280 nm) using a NanoDrop® 1000 (Thermo Scientific) and then diluted to 10 ng/μL. DNA purified and adjusted solutions were subsequently stored at −20°C and warm to room temperature immediately prior to use.

### PCR amplification

A PCR amplification assay was initially performed using the Illustra Ready-To-Go PCR Beads (GE Healthcare) in a 25 μL total reaction volume containing 2 μL of DNA template (Sonora sample) (10 ng/μL), 1 μL of each primer (10 pmol) and 23 μL of nuclease free water. Cycling conditions for the amplification were: 95°C for 5 min, followed by 30 cycles of 94°C/30 s, 61°C/30 s, and 72°C/30 s and a final extension step of 72°C for 5 min. Amplification products were electrophoresed in a 1.2% agarose gel, and visualized and documented under UV light using a KODAK Gel Logic 100 Imaging System (Eastman Kodak). The WSSV VP28 amplicon was carefully excised from the gel, purified using the commercial Illustra GFX™ PCR DNA and Gel Band Purification Kit (GE Healthcare) according to manufacturer’s protocol, and diluted to a 1.88 ng/μL working concentration. Ten-fold serial dilutions with known copy numbers ranging from 1.24 × 10^7^ to 12 copies/μL were then prepared from the purified target amplicon stock. The concentration in each dilution was converted to copy numbers by using the following equation:

$$\begin{array}{ll} \mathrm{Number}\;\mathrm{of}\;\mathrm{copies}= & \left(\mathrm{mass}\times 6.022\times 10^{23}\right)/\\ & \left(\mathrm{length}\times 10^{9}\times 650\right). \end{array}$$

Where the mass is the amount of DNA in nanograms, length is the size of the amplicon in base pairs, and the average weight of a base pair is assumed to be 650 Da.

### Quantitative real-time PCR (qPCR) assay: efficiency, linearity and precision

For the initial qPCR, reactions were performed in a 14 μL volume comprised of 1 μL of DNA of each template dilution, 7.5 μL of 2× iQ SYBR® Green supermix (Biorad), and 0.5 μL of each primer (10 pmol), and 4.5 μL of nuclease free water. Real-Time PCR assays were performed in a Rotor-Gene 3000 real-time rotary thermal cycler (Corbett Life Science), and cycling parameters were: 95°C for 5 min, followed by 30 cycles of 94°C/30 s, 61°C/30 s, and 72°C/30 s and a final extension step of 72°C for 5 min. Fluorescence measurements (510 nm) were taken at the end the elongation phase for each cycle. Melting curve analysis, to detect the occurrence of primer-dimer formation or the amplification of other non-specific products, were performed immediately after amplification by slow heating of the samples from 60 to 99°C with a 0.3°C/s ramping rate and stepwise fluorescence detection at 0.3°C interval. The melting curves were converted to melting peaks by plotting the negative derivatives of fluorescence against temperature (−dF/dT). All reactions were done in triplicate, and the quantification cycle (C_q_) values were automatically calculated with the Rotor-Gene software version 6.1. The qPCR reaction efficiency and linearity were calculated from the standard curve.

To determine the precision of the qPCR assay, aliquots of five serial dilutions of the stock DNA (Sonora sample) were assayed in triplicate two times over a 2 months period and precision was determined by calculating the coefficient of variation (CV).

In addition, in order to compare the assay precision, qPCR was also conducted with the dilution DNA samples containing 1.24 × 10^8^ and 1.24 × 10^2^ copies/μL in triplicate using a different detection chemistry kit, the GoTaq® qPCR Master mix (Promega). Samples were prepared as above and run in a Rotor-Gene 3000 Real-Time PCR system. Finally, the precision of the assay was also assessed by running a qPCR test with aliquots of the DNA samples from different geographical locations by different skilled members of the CIBNOR staff.

### Primer specificity and sensitivity

The specificity of the primers for the detection of a fragment of the WSSV VP28 gene was initially evaluated *in silico* using the BLAST nucleotide software against representative sequences of the complete genome of 6 shrimp infecting viruses (YHV, tax ID number: 96029; TSV, tax ID number: 142102; IHHNV, tax ID number: 11792; PvNV, tax ID number: 430911; IMNV, tax ID number: 282786; and WSSV, tax ID number: 342409).

In order to evaluate the specificity of the qPCR assay developed in this study, a panel of 6 shrimp infecting viral isolates (Yellow Head Virus, YHV; Taura Syndrome Virus, TSV; Infectious Hypodermal and Hematopoietic Necrosis Virus, IHHNV, *Penaeus vannamei* Nodavirus, *Pv*NV; Infectious Mionecrosis Virus, IMNV; and WSSV), obtained through an inter-laboratory calibration test implemented by an OIE reference laboratory, were used as PCR templates. Reaction mixtures for qPCR were prepared as described above.

### Amplification of WSSV samples from different geographical locations and detection on a wide range of crustaceans

The universality of this primer pair was tested by the amplification of a WSSV VP28 fragment from a number of geographic locations. Reaction mixtures and cycling conditions for qPCR were performed as above described.

The primers VP28-140Fw and VP28-140Rv were tested against DNA of 10 different species of WSSV-infected crustaceans. The qPCR reactions were carried out as described above.

## Competing interests

The authors declare that they have no competing interests.

## Authors’ contributions

FMC and ASP designed research. FMC carried out the molecular analysis. ASP conceived the study, analyzed data and drafted the manuscript. All authors have read and approved the final manuscript.
